# Exploration into the origins and mobilization of di-hydrofolate reductase genes and the emergence of clinical resistance to trimethoprim

**DOI:** 10.1099/mgen.0.000440

**Published:** 2020-09-24

**Authors:** Miquel Sánchez-Osuna, Pilar Cortés, Montserrat Llagostera, Jordi Barbé, Ivan Erill

**Affiliations:** ^1^​ Departament de Genètica i de Microbiologia, Universitat Autònoma de Barcelona, Bellaterra, Spain; ^2^​ Department of Biological Sciences, University of Maryland, Baltimore County, Baltimore, MD, USA

**Keywords:** trimethoprim, antibiotics, chemotherapeutic agent, resistance, sulfonamides, phylogenetics, evolution

## Abstract

Trimethoprim is a synthetic antibacterial agent that targets folate biosynthesis by competitively binding to the di-hydrofolate reductase enzyme (DHFR). Trimethoprim is often administered synergistically with sulfonamide, another chemotherapeutic agent targeting the di-hydropteroate synthase (DHPS) enzyme in the same pathway. Clinical resistance to both drugs is widespread and mediated by enzyme variants capable of performing their biological function without binding to these drugs. These mutant enzymes were assumed to have arisen after the discovery of these synthetic drugs, but recent work has shown that genes conferring resistance to sulfonamide were present in the bacterial pangenome millions of years ago. Here, we apply phylogenetics and comparative genomics methods to study the largest family of mobile trimethoprim-resistance genes (*dfrA*). We show that most of the *dfrA* genes identified to date map to two large clades that likely arose from independent mobilization events. In contrast to sulfonamide resistance (*sul*) genes, we find evidence of recurrent mobilization in *dfrA* genes. Phylogenetic evidence allows us to identify novel *dfrA* genes in the emerging pathogen *Acinetobacter baumannii *, and we confirm their resistance phenotype *in vitro*. We also identify a cluster of *dfrA* homologues in cryptic plasmid and phage genomes, but we show that these enzymes do not confer resistance to trimethoprim. Our methods also allow us to pinpoint the chromosomal origin of previously reported *dfrA* genes, and we show that many of these ancient chromosomal genes also confer resistance to trimethoprim. Our work reveals that trimethoprim resistance predated the clinical use of this chemotherapeutic agent, but that novel mutations have likely also arisen and become mobilized following its widespread use within and outside the clinic. Hence, this work confirms that resistance to novel drugs may already be present in the bacterial pangenome, and stresses the importance of rapid mobilization as a fundamental element in the emergence and global spread of resistance determinants.

## Data Summary

- Nucleotide and protein sequences analysed in this study have been downloaded from publicly available National Center for Biotechnology Information databases.
- The scripts used for data collection and analysis can be obtained at the GitHub ErillLab repository (https://github.com/ErillLab/).
- The Bayesian phylogenetic tree can be visualized online on iTOL (https://itol.embl.de/tree/855674159451585133078) [1].

**Impact Statement**

Antibiotic resistance is a pressing and global phenomenon. It is well established that resistance to conventional antibiotics emerged millions of years ago in either antibiotic-producing bacteria or their competitors. Resistance to synthetic chemotherapeutic agents cannot be explained by this paradigm, since these drugs are not naturally produced. Hence, resistance is assumed to have evolved rapidly following the clinical introduction of these drugs. Recently, we showed that resistance to one such drug, sulfonamide, evolved not recently, but millions of years ago, suggesting that the diversity of bacterial genomes may well contain genes conferring resistance to drugs yet to be developed. Here, we analyse the origin of resistance to trimethoprim, another chemotherapeutic agent developed in the 1960s. Using phylogenetic methods, we identify new variants of the trimethoprim-resistance genes that had not previously been reported, and we trace the chromosomal origins for a number of already known resistance variants. Our results show that resistance to trimethoprim is very diverse, and has originated both from recent mutations and from pre-existing ancient variants. These results stress the importance of gene mobilization mechanisms as the main drivers of the current antibiotic-resistance phenomenon.

## Introduction

Bacterial resistance to antibacterial agents remains an increasingly challenging and global problem in modern health care [2, 3]. Bacterial cells display a diverse array of mechanisms to cope with exposure to antibacterial compounds, including modification or overexpression of the antibacterial target, efflux or reduction of antibacterial uptake and the use of alternate pathways [4]. Constant exposure to non-lethal concentrations of antibacterial agents may lead to the selection of partial resistance to antibiotics over relatively short time spans [5], and this evolution may be hastened by simultaneous exposure to multiple antibacterials [6]. However, the rapid proliferation of multidrug-resistant nosocomial pathogens in the last 50 years has not been driven by the independent evolution of resistance traits, but through the extensive dissemination of mobile genetic elements carrying resistance genes [4, 7]. It is widely accepted that most genes conferring resistance to antibiotics present in pathogenic bacteria were obtained by successive lateral gene transfer of homologues that originally evolved in the microbes that produce the antibiotic or in their natural competitors [7, 8]. The high plasticity of bacterial genomes, enabled by a large repertoire of mobile genetic elements, and the availability of a large pool of ancient antibiotic-resistance determinants, hence, set the stage for the rapid proliferation of antibiotic resistance, giving rise to multi-resistant clinical strains just a few years after the commercial introduction of antibiotics [7].

Synthetic chemotherapeutic agents predate antibiotics in the clinical setting, and continue to be used synergistically with antibiotics to treat microbial infections [9]. Following the initial discovery and clinical use of arsphenamine in 1907 [10], interest in chemotherapeutic agents quickly took off after the development of sulfa drugs in the 1930s [11]. The discovery of trimethoprim (a di-aminopyrimidine) was received with interest because, like sulfonamides, trimethoprim targets the bacterial synthesis of tetrahydrofolic acid, which is a necessary cofactor in the synthesis of thymine and purines [12]. Sulfonamides are structural analogues of para-aminobenzoic acid (PABA) and inhibit the synthesis of di-hydropteroate by competing with PABA for binding to the di-hydropteroate synthase (DHPS) enzyme, resulting in sulfonamide-bound di-hydropterin [13]. Trimethoprim is a structural analogue of di-hydrofolic acid, derived from di-hydropteroate. It acts by competitively binding to the di-hydrofolate reductase (DHFR) enzyme; hence, inhibiting the production of tetrahydrofolic acid [13, 14]. The synergistic use of trimethoprim and sulfonamides was expected to have a potent bactericidal effect by producing a serial blockade on the tetrahydrofolic acid pathway [12, 15].

Unlike antibiotics, chemotherapeutic agents are not produced by natural organisms, yet resistance to these novel drugs arose quickly after their mass-production and it is today pervasive among clinical isolates [7]. In the case of sulfonamides and trimethoprim, which are usually administered in tandem, resistance via chromosomal mutations to both chemotherapeutics was reported soon after their clinical introduction [13]. Chromosomal resistance to sulfonamides can occur through mutations yielding increased production of PABA [16] or, more commonly, via mutations to the chromosomal *folP* gene (encoding DHPS), which decrease the affinity of DHPS for sulfonamide without detriment to PABA binding [13, 17]. Such mutations have been reported in multiple bacterial groups and target different conserved regions of DHPS [13]. Similarly, chromosomal resistance to trimethoprim may arise via mutations that increase transcription of the *folA* gene (encoding DHFR) [18], or through mutations that decrease the affinity of DHFR for trimethoprim [13]. The vast majority of resistant clinical isolates to both sulfonamides and trimethoprim, however, are not due to chromosomal mutations, but to the acquisition of resistance determinants on mobile genetic elements [13]. Parallel to their systematic combined use in both clinical and agricultural settings, genes conferring resistance to sulfonamides and trimethoprim are frequently found together on mobile elements, such as class 1 integrons [19] or conjugative plasmids [13, 20]. The mobile genes conferring resistance to sulfonamide are homologues of the chromosomally encoded *folP* gene and are collectively known as *sul* genes (for *sul*fonamide resistance). Mobile genes conferring resistance to trimethoprim are either homologues or functional analogues of the chromosomally encoded *folA* gene and are collectively known as *dfr* genes (for *d*i-hydro*f*olate *r*eductase) [17].

In spite of their frequent co-occurrence on mobile genetic elements, there are significant differences between the mobile genes conferring resistance to sulfonamides (*sul* genes) and trimethoprim (*dfr* genes). To date, only three *sul* gene classes have been described in clinical isolates [21], whereas more than 30 different *dfr* genes have been reported in clinically relevant strains [22]. Trimethoprim-resistance (*dfr*) genes have been further classified into two families (*dfrA* and *dfrB*). These two families encode evolutionarily unrelated proteins of markedly different sizes. Sequence similarity indicates that *dfrA* genes are homologues of the chromosomally encoded *folA* genes, whereas *dfrB* genes are functional analogues of unknown origin [23, 24]. Most *dfrA* genes follow a standard naming convention consisting of *dfrA* followed by a numerical value indicating their discovery rank order. However, several *dfrA* genes first identified in Gram-positive bacteria, and thought at the time to be unrelated to the Gram-negative *dfrA* genes, were originally named following an alphabetical convention (*dfrC–K*). The disparity in genetic diversity among sulfonamide and trimethoprim mobile resistance determinants is suggestive of different evolutionary processes leading to the onset and spread of resistance to these two chemotherapeutic agents [13]. It was suggested that resistance to sulfonamide had arisen in a few isolated organisms and rapidly spread upon the introduction of sulfa drugs, whereas trimethoprim resistance had independently evolved, and had been subsequently mobilized multiple times [13].

Recently, we examined the origins of *sul* genes through comparative genomics, phylogenetic analysis and *in vitro* assays [25]. We identified a well-defined mutational signature in *sul*-encoded proteins with respect to chromosomally encoded *folP* genes, and we used this conserved motif to map the origins of *sul* genes in bacterial chromosomes. Our work revealed that the three groups of *sul* genes identified in clinical isolates originated in the *Leptospiraceae* and were transferred to the *Rhodobiaceae* more than 500 million years ago. These two ancient resistant determinants were later independently mobilized, and rapidly disseminated following the commercial introduction of sulfa drugs. By tracing the phylogenetic lineage of *sul* genes and demonstrating that these two bacterial families were resistant to sulfonamides long before their discovery and clinical use, our work indicated that resistance to novel drugs could very well pre-exist, and be ready for mobilization, within the vast bacterial pangenome. Here, we apply similar methods to examine the phylogenetic relationships among *dfrA* and chromosomally encoded *folA* genes. Our aim is to shed light on the evolutionary processes giving rise to mobile trimethoprim-resistance genes. Our work illustrates significant similarities and differences in the processes leading to the emergence and spread of trimethoprim- and sulfonamide-resistance determinants, reveals previously unreported clusters of *dfrA* genes, and suggests that systematic analyses of the bacterial pangenome may be of use in the design of novel antibacterials.

## Methods

### Sequence data collection

To identify homologues of DfrA proteins, we first compiled a panel of Dfr proteins reported in the literature. Dfr proteins belong to two distinct families of unrelated sequences (DfrA and DfrB; Fig. S1, available with the online version of this article). We mapped these sequences to pfam models of DfrA (PF00186) and DfrB (PF06442) (Table S1) using hmmer version 3.1b2 (hmmscan) [26], and we discarded sequences mapping to the DfrB family, retaining only DfrA proteins for analysis (Table S2). We further excluded redundant DfrA sequences (amino acid sequence identity >90 %) using t-coffee version 11.00.8cbe486 seq_reformat command [27], and used the resulting non-redundant panel to identify DfrA homologues in protein records associated with National Center for Biotechnology Information (NCBI) GenBank/RefSeq sequences corresponding to mobile genetic elements. These were defined as sequences containing the keywords ‘plasmid’, ‘integron’ or ‘transposon’ in their title, belonging to complete genome records [28, 29]. Protein records corresponding to blastp hits matching stringent value (<1e^−20^) and query coverage (>75 %) thresholds were added to the panel if non-redundant (amino acid sequence identity <90 % with respect to existing panel members), and their classification as mobile elements was validated by assessing that the nucleotide record encoding them contained at least one gene encoding an integrase, transposase or plasmid replication protein, as determined by hmmer (hmmscan, *E* value <1e^−05^) with reference pfam models (Table S3) [30–34]. To detect putative chromosomally encoded *folA* genes associated with mobile *dfrA* genes, we used the sequences in the extended non-redundant panel of DfrA homologues as queries for tblastn searches against NCBI GenBank complete genomes with stringent *E* value (<1e^−40^) and query coverage (>75 %) settings. Hits with nearby genes annotated as resistance determinants, transposases or integrases were considered to encode chromosomally integrated mobile DfrA homologues and not considered for further analysis. For each mobile DfrA homologue in the panel, the first, if any, tblastn hit satisfying these thresholds was considered, for the purposes of this study, to be a proxy for the closest putative chromosomally encoded FolA protein. The choice of representative DfrA sequences did not alter the tblastn results. To complete the panel of protein sequences used to reconstruct the evolutionary history of DfrA/FolA sequences, we used the non-redundant panel of mobile DfrA sequences to identify via blastp (*E* value <1e^−20^, coverage >75 %) FolA proteins encoded by the chromosomes of NCBI RefSeq representative species for all bacterial orders, and for each bacterial family in the *Proteobacteria *. In addition, the closest archaeal homologues of bacterial FolA sequences were determined by searching with blastp the NCBI protein database, restricted to *Archaea* (taxid:2157), with the *Escherichia coli* FolA protein. A member of each family from the order (* Halobacteriales *) of the identified best archaeal hit of *E. coli* FolA was sampled to populate the outgroup.

### Phylogenetic analysis

For phylogenetic inference, we performed a t-coffee multiple sequence alignment of protein sequences for the complete panel of DfrA and FolA homologues, combining three clustalw (version 2.1) profile amino acid sequence alignments with different (5, 10, 25) gap opening penalties and leveraging the *E. coli* FolA crystal structure (P0ABQ5) to adjust gap penalties [35]. The resulting amino acid sequence alignment was processed with Gblocks version 0.91b (allowed gap positions, with half; minimum number of sequences for a conserved position, 86; minimum number of sequences for a flanking position, 95; maximum number of contiguous nonconserved positions, 5; minimum length of a block, 4) [36]. Bayesian phylogenetic inference on the trimmed multiple amino acid sequence alignment was carried out with MrBayes version 3.2.6 [37]. Four Metropolis-coupled Markov chain Monte Carlo simulations with four independent chains were run for 20 000 000 generations, using a mixed four-category gamma distributed rate plus proportion of invariable sites model [invgamma] and a JTT (Jones–Taylor–Thornton) amino acid substitution model [38]. Chains were sampled every 100 iterations and stationarity was analysed with Tracer version 1.7.1 [39] by monitoring the estimated sample size (ESS). To determine burn-in, chain results were summarized with MrBayes imposing the restriction that ESS be above 200 and that the potential scale reduction factor be within 0.005 of 1. Based on summarization results, the burn-in was set at 20 % of iterations. A consensus tree was generated with the half-compat option and visualized using the ggtree version 1.14.6 R library [40]. Clades of reported DfrA proteins were determined as branches with posterior probability values higher than 0.8 containing at least five reported DfrA sequences. Ancestral state reconstruction of a single binary trait (mobile/chromosomal) was performed with BayesTraits version 3.0.2 [41]. The mobile/chromosomal state of each sequence was determined through the data collection pipeline outlined above. Known states at tree tips were labelled, and ancestral states were reconstructed using the multistate and maximum-likelihood settings.

### DNA techniques and *in vitro* trimethoprim-susceptibility assay

With the exception of the *Ralstonia solanacearum* GMI1000 (Marc Valls, Center for Research in Agricultural Genomics, Barcelona, Spain) and *E. coli* K-12 (CGSC5073) *folA* genes, which were amplified from genomic DNA, *dfrA* and *folA* homologues were adapted to *E. coli* codon usage, synthesized (ATG:biosynthetics) and then subcloned into a dephosphorylated pUA1108 vector [42] using an *Nde*I and *Bam*HI (New England Biolabs) double digest when possible. Genes with internal restriction sites for any of these two enzymes were subcloned into the same vector using the HiFi DNA assembly kit (New England Biolabs), following the manufacturer’s protocol. Oligonucleotides used in this work are listed in Table S4. All constructs were validated by sequencing (Macrogen) prior to their use in transforming *E. coli* K-12 (CGSC5073). The minimum inhibitory concentration (MIC) for trimethoprim (Sigma-Aldrich) for strains of *E. coli* K-12 (CGSC5073) carrying different versions of pUA1108 encoding *folA* or *dfrA* homologues was determined following the Clinical and Laboratory Standards Institute guidelines using microdilution tests in Mueller–Hinton (MH) broth (Merck) [43]. All MIC assays were performed in triplicate. Colonies were grown on Luria–Bertani agar for 18 h and then suspended in sterile 0.9 % NaCl solution to a McFarland 0.5 turbidity level. Suspensions were then diluted at 10^−2^ in MH broth, and 50 µl (5×10^4^) cells were inoculated into microtitre plates that contained 50 µl MH broth supplemented with 1024–0.250 mg trimethoprim l^−1^. To determine growth, optical density at 550 nm was measured after 24 h incubation at 37 °C. The *dfrA1* gene was used as a positive control [44] and the *E. coli folA* gene as a negative control [45].

### Sequence analysis

To assess whether the identified chromosomal gene associated with a mobile *dfrA* gene is the canonical *folA* gene for the genus, and not the product of a subsequent recombination of the mobile *dfrA* gene into the chromosome, we computed the pairwise amino acid identity among the products of all chromosomal *folA* homologues and then compared this distribution with the pairwise amino acid identity of the putative origin versus the chromosomal *folA* homologues. We used a one-sided Mann–Whitney U test to determine whether the two distributions were significantly different. To analyse the mol% G+C content relationship between *sul*/*dfrA* genes and their host chromosomes, we used pre-compiled panels of sequences for non-redundant Sul [25] and DfrA homologues to search protein records associated with NCBI GenBank/RefSeq sequences of mobile genetic elements. The nucleotide sequences of the genes encoding these proteins was then retrieved. The mol% G+C content of the corresponding *sul* and *dfrA* genes, as well as the overall mol% G+C content in both the mobile genetic element and the chromosome of the species harbouring it, were computed with custom Python scripts. To analyse whether mobile *dfrA* genes with mol% G+C content close to their hosts’ genomes are more similar to the hosts’ *folA* genes than expected if *dfrA*–host associations were arbitrary, we performed a permutation test comparing the mean pairwise amino acid sequence alignment distance between DfrA proteins and host-encoded FolA proteins. We randomly permuted DfrA-host assignments 1000 times and computed the corresponding *P* value as the rank of the non-permuted mean pairwise alignment distance. The input files and scripts used for data collection and analysis are available in public repositories: (i) input files (json, txt and fasta) and blast database for Python scripts used in data collection and analysis – https://doi.org/10.6084/m9.figshare.12156891.v1, and (ii) GitHub repository containing the Python scripts used for data collection and analysis – https://doi.org/10.5281/zenodo.3760352.

## Results and Discussion

### A large fraction of reported *dfrA* genes share a common evolutionary origin

To explore the phylogenetic relationship of trimethoprim-resistance determinants with their chromosomally encoded *folA* counterparts, we used a non-redundant panel of protein sequences encoded by reported *dfr* genes (Table S2) to detect putative DHFR homologues in sequenced mobile elements. We discarded sequences associated with the *dfrB* gene family, and retained for analysis non-redundant sequences mapping to the clades defined by *dfrA* genes reported in the *Proteobacteria* and by *dfrDEFGK* genes associated with Gram-positive bacteria. For convenience, and in accordance with recent reports on *dfr* nomenclature [46], we hereinafter designate these two groups (*dfrA* and *dfrDEFGK*) with the umbrella term *dfrA*. These reference mobile DfrA homologues were then combined with FolA homologues sampled from representative genomes of all bacterial orders with complete genome assemblies, and of each bacterial family within the *Proteobacteria* (Table S5). The resulting multiple amino acid sequence alignment was used to perform Bayesian phylogenetic analysis of FolA/DfrA sequences.

**Fig. 1.**
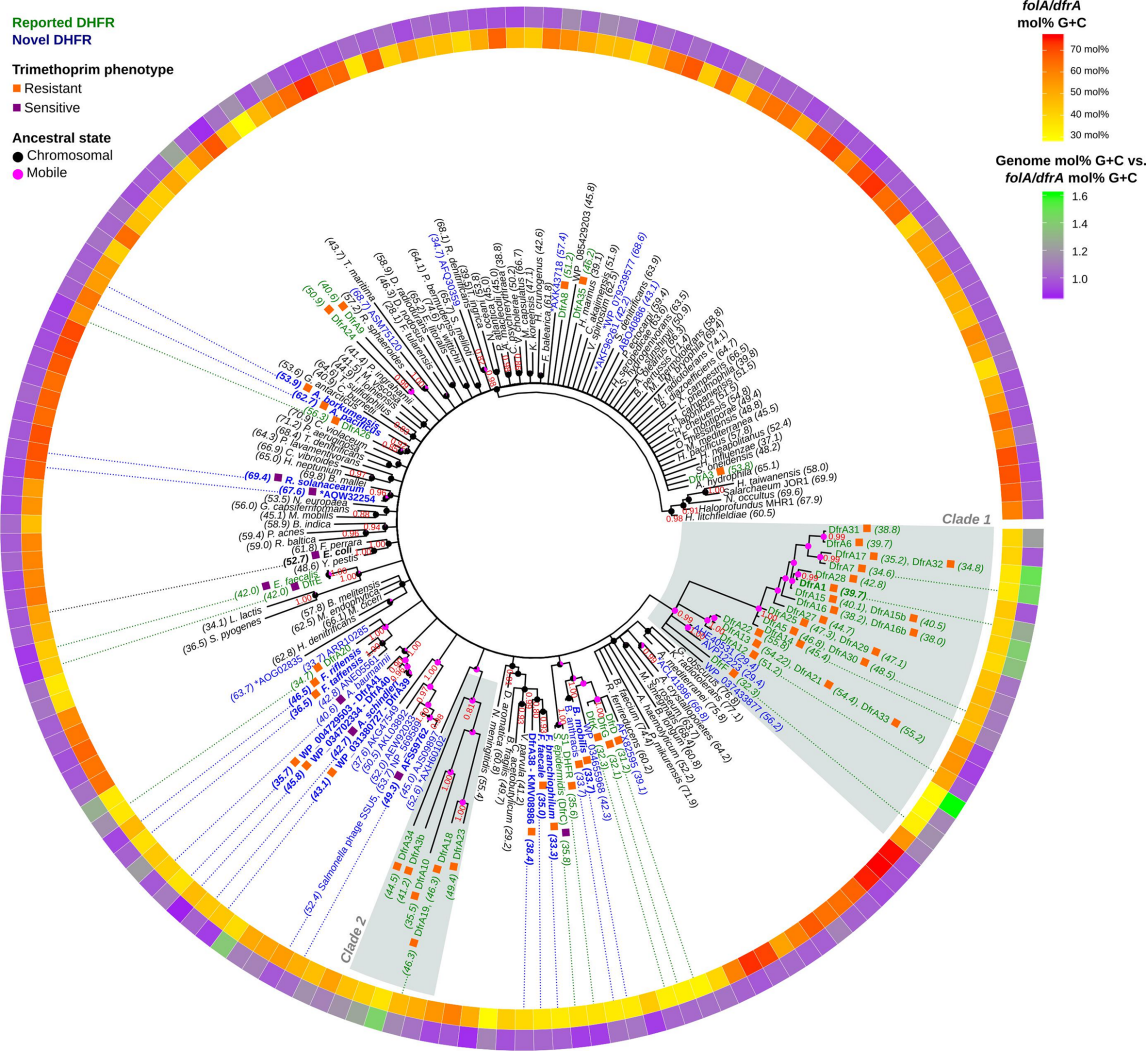
Consensus tree of DHFR protein sequences. Branch support values are provided as Bayesian posterior probabilities estimated after four independent runs of 20 000 000 generations. Support values are only shown for branches with posterior probability values higher than 0.8. For chromosomal DHFR, the species name is displayed. Mobile DHFRs are denoted by their established *dfrA* name or by their NCBI GenBank accession number. Reported *dfrA* genes deemed redundant (>90 % identity) are listed next to the corresponding non-redundant taxon included in the analysis. Next to each tip label, coloured boxes designate trimethoprim-resistant (orange) and -sensitive (purple) DHFR. Numbers between brackets indicate the mol% G+C content of the sequence for the gene encoding the DHFR. Tip label colouring denotes previously reported (green) and novel (blue) DHFRs. Bold label text indicates that resistance has been experimentally assessed in this work. DHFR variants marked with an asterisk are encoded in megaplasmids (>400 kbp). The internal ring shows the mol% G+C of the gene encoding the DHFR in a yellow−red colour scale, while the external ring displays the ratio between the mol% G+C content of the genome harbouring the DHFR gene and the mol% G+C content of the gene. Dotted lines from the inner ring to tip labels denote genes discussed in the text. Reconstructed mobile/chromosomal states are displayed on ancestral nodes as pink/black pie charts.

The phylogenetic tree shown in Fig. 1 showcases the genetic diversity of DfrA/FolA proteins, which encompass sequences with pairwise amino acid sequence identities ranging from 99 to 20 % (Table S6). The resulting phylogeny also reveals that the vast majority (70.7%) of known DfrA sequences map to two well-supported (>0.8 posterior probability), distinct clades that likely arose from two different ancestors. The first clade (clade 1), typified by the DfrA1 and DfrA12 proteins [47], includes 22 sequences encoded by previously reported *dfrA* genes with a mean amino acid identity of 51.19 % ±17.63 sd, divided into two subgroups (containing 17 and 5 known *dfrA* genes, respectively) and associated with *Gammaproteobacteria* pathogens. This clade also includes a basal set of taxa composed of the *Clostridioides difficile dfrF* gene and two newly identified mobile *dfrA* homologues also from *Firmicutes* isolates. The second clade (clade 2), exemplified by DfrA18 [48], comprises a group of six highly diverged (34.37%±10.15 sd mean amino acid identity) DfrA sequences from *Gammaproteobacteria* isolates.

Analysis of the *dfrA* gene sequences in these two clades reveals an unexpected degree of heterogeneity in mol% G+C content. In the first clade, several *dfrA* homologues, including the *C. difficile dfrF* gene, show relatively low mol% G+C content (Fig. 1, inner ring), matching the *Firmicutes* species they were reported on (Table S7, Fig. 1, outer ring). Similarly, *dfrA* genes in the *dfrA12* group show a mol% G+C content (53.28 mol%±1.80 sd) that is well in line with that of the *Enterobacteriaceae* isolates harbouring them. Conversely, the largest group in this clade, encompassing *dfrA1*, *dfrA7* and *dfrA14*, shows a mean mol% G+C content of 41.03 mol%±3.99 sd, which is substantially lower than the average mol% G+C content of the *Enterobacteriaceae* harbouring these mobile elements. The same holds true for the second clade (*dfrA18*), which also shows lower mol% G+C content (43.88 mol%±4.91 sd) than expected for the *Enterobacteriaceae *. To ascertain whether this pattern of mol% G+C heterogeneity extended to other previously reported and putative *dfrA* genes, we examined the mol% G+C content of *dfrA* (935 genes) and *sul* (408 genes) homologues identified in this analysis with respect to the genome mol% G+C content of the host species harbouring these mobile resistance genes.

**Fig. 2.**
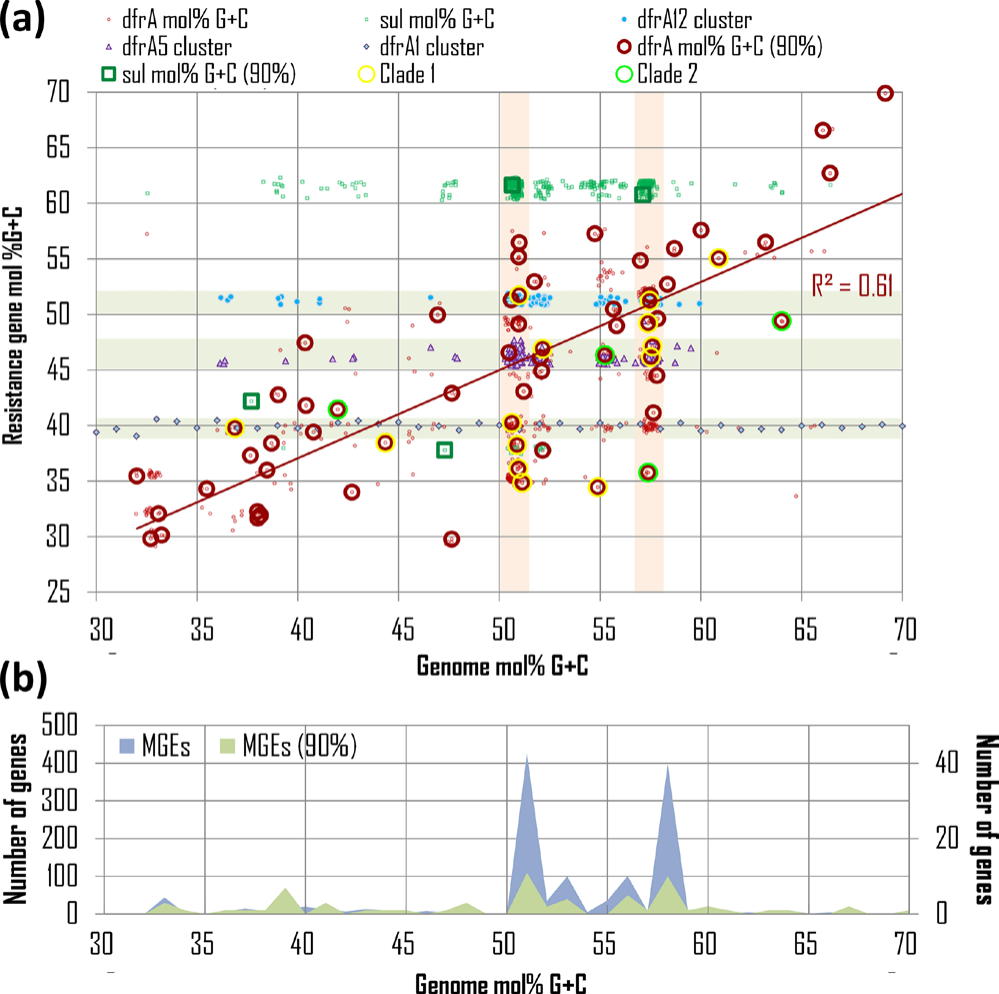
Correlation between the mol% G+C content of mobile *dfrA* (red circles) and *sul* (green squares) genes and that of their host genome. Large open circles/squares denote representatives of clusters of redundant sequences (identity >90 %), and *dfrA* genes from clade 1 and clade 2 in Fig. 1 are marked with an additional corona. A 0.75 % jitter to both *x*- and *y*-axis values has been applied for visualization purposes. The red line shows the linear regression for representative *dfrA* gene values. The Pearson R^2^ coefficient is superimposed. Vertical background bars in (a) designate DfrA sequences harboured by mobile genetic elements (MGEs) identified in *E. coli* and *K. pneumoniae* isolates, which are heavily overrepresented in the dataset. Sequences from clusters with more than 100 sequences (represented by *dfrA12*, *dfrA5* and *dfrA1*) are shown with specific markers, and highlighted by horizontal background bars. The number of MGEs identified as harbouring *dfrA* genes, before and after filtering DfrA sequence identity (>90 %), is shown in (b).

The results shown in Fig. 2(a) and Table S8 reveal that *dfrA* genes tend to align with host genome mol% G+C content (Pearson ⍴=0.56), whereas *sul* genes display a two-tiered distribution of mol% G+C content that is essentially independent of host genome mol% G+C (Pearson ⍴=0.14). Available *dfrA* and *sul* sequences are dominated by variants of a known *dfrA* and *sul* genes that have been isolated predominantly in a select group of bacterial hosts (Fig. 2b). To correct for this skew, we filtered *dfrA* sequences based on the amino acid identity (<90 %) of their encoded proteins. This filtering resulted in a significantly smaller number of non-redundant representative *dfrA* (63 genes) and *sul* (4 genes) sequences (Table S9). The four representative *sul* genes correspond to one exemplar of the *sul1* and *sul2* families, and two exemplars of the *sul3* family. Among representative *dfrA* genes, 14 map to the first clade (clade 1) of Fig. 1 and 4 to the second clade (clade 2). The correlation of *dfrA* genes with host genome mol% G+C increases significantly (Pearson ⍴=0.78) when considering only non-redundant representative *dfrA* sequences. The fact that the mol% G+C content of representative *dfrA* sequences aligns well with their host genome mol% G+C could suggest that mol% G+C content in *dfrA* genes has been ameliorated to match the host’s. Alternatively, it could indicate that the mobile *dfrA* gene originated via mobilization of a chromosomal *folA* gene from a bacterium in the same clade as the current host. The later scenario posits that, besides mol% G+C content similarity, representative *dfrA* genes should also encode proteins with significant sequence similarity to their hosts’ FolA protein. We performed a permutation test to analyse whether representative *dfrA* gene products show significant similarity with their hosts’ FolA protein (Table S10). Our results indicate that this is the case (*P*<0.001), suggesting that most mobile *dfrA* genes are still associated with species from the same clade they originated in.

The filtering of *dfrA* sequences based on amino acid identity brings forward three large clusters (represented by *dfrA12*, *dfrA5* and *dfrA1*, and belonging to clade 1 from Fig. 1) containing more than 100 genes with amino acid identity larger than 90 %. The *dfrA* genes in these clusters show a distribution of mol %G+C content that is essentially independent of the host genome mol% G+C, as in the case of *sul* genes (Fig. 2a), and their products show no significant sequence similarity with the hosts’ FolA (permutation test *P*>0.1). This indicates that the *dfrA* genes in these large clusters have spread across distantly related bacterial clades, primarily through their association with *sul*-containing integron-based transposable elements that are widely disseminated among clinically relevant bacteria [49, 50]. This analysis also brings to the fore the presence of multiple *dfrA* cluster representatives, with widely divergent mol% G+C, on narrow bands of host genome mol% G+C content. These bands correspond to *E. coli* (50.7 mol% G+C) and *Klebsiella pneumoniae* (57.4 mol% G+C) isolates, which are heavily oversampled in the dataset (Fig. 2b). The marked divergence in mol% G+C content (* E. coli *, 9.55 mol %±6.15 sd; *K. pneumoniae *, 6.93 mol %±4.63 sd) and amino acid sequence identity (* E. coli *, 38.57 % ±15.24 sd; *K. pneumoniae *, 38.39 % ±14.02 sd; Table S11, Fig. S2) among these representative *dfrA* genes suggests that they originated via mobilization from a diverse set of chromosomal backgrounds.

The fact that the mol% G+C of *dfrA* genes aligns with their host genome’s mol% G+C, and that DfrA proteins display higher amino acid sequence identity when aligned to their host genome FolA proteins than to other FolA proteins, strongly supports the notion that *dfrA* genes have been mobilized multiple times within different bacterial clades [13]. In a few instances, typified by the large *dfrA* clusters illustrated in Fig. 2, *dfrA* genes have been captured by highly efficient mobile elements and dispersed widely across unrelated groups of bacteria [49]. These mobile elements often harbour *sul* genes, which also display a host-independent mol% G+C distribution. Many of the *dfrA* genes identified here are associated with clinical isolates. The divergent mol% G+C content and amino acid identity of these *dfrA* genes indicates that pathogenic bacteria have obtained *dfrA* genes on multiple occasions and from different sources, highlighting the ability of mobilized resistance determinants to reach clinically relevant pathogens [17, 51].

### Novel trimethoprim-resistance determinants of *Acinetobacter* clinical isolates identified through phylogenetic methods

The phylogenetic tree in Fig. 1 includes reported DfrA proteins and their putative homologues, as well as FolA proteins identified via tblastn as putative DfrA homologues or sampled uniformly across bacterial clades. The inferred phylogeny also reveals several groups of previously unreported mobile DHFR homologues that form well-supported clades in association with chromosomal FolA proteins. Hence, these FolA proteins could constitute the chromosomal origins of the associated mobile DHFR homologues, and provide insights into the emergence and dissemination of trimethoprim-resistance genes. To determine whether these mobile DHFR homologues did confer resistance to trimethoprim, we cloned a subset of *dfrA*/*folA* genes and performed broth microdilution assays to determine the MIC of trimethoprim. Considering that the clinical breakpoint for trimethoprim in *E. coli* is 4 mg l^−1^ [52], the results, shown in Table 1, reveal that most of the mobile DHFR homologues identified here do confer significant resistance to trimethoprim. The sole exception is the protein AQW32254. Close inspection revealed that this DHFR homologue is encoded by a megaplasmid (1.2 Mb) from a *Ralstonia* isolate, and that this is the only DHFR homologue present in its complete genome. Hence, we determined that this DHFR homologue was a bona fide FolA protein and not a mobile DHFR homologue, and we excluded from further analysis all other DHFR homologues identified in megaplasmids (>400 kbp).

**Table 1.** MICs of trimethoprim for wild-type *E. coli* K-12 (CGSC5073) and derivatives carrying different versions of *dfr*/*folA* or the control empty vector Values are representative of four independent replicates.

Strain	Mobile / chromosomal	Nucleotide accession no.	Cloned protein ID	Trimethoprim (mg l^−1^)
* E. coli* CGSC5073	–	–	–	0.25
* E. coli* pUA1108	–	–	–	0.25
* E. coli* pUA1108::*folA E. coli*	C	NC_000913	WP_000624375	4
* E. coli* pUA1108::*dfrA1*	M	NC_002525	WP_000777554	>512
* E. coli* pUA1108::*folA Flavobacterium branchiophilum*	C	NC_016001	WP_014083133	256
* E. coli* pUA1108::*folA Flavobacterium faecale*	C	NZ_CP020918	WP_108740183	>512
* E. coli* pUA1108::*dfrA38 Acinetobacter baumannii*	M	CP021344	KMV08986	256
* E. coli* pUA1108::*folA Acinetobacter schindleri*	C	NZ_CP025618	WP_004813248	0.25
* E. coli* pUA1108::*dfrA39 Acinetobacter baumannii*	M	NZ_CP021785	WP_031380727	512
* E. coli* pUA1108::*dfrA40 Acinetobacter baumannii*	M	NZ_JEVW01000010	WP_034702334	128
* E. coli* pUA1108::*dfrA41 Acinetobacter defluvii*	M	NZ_CP029396	WP_004729503	>512
* E. coli* pUA1108::*folA Fluviicola taffensis*	C	NC_015321	WP_013685591	>512
* E. coli* pUA1108::*folA* 'Candidatus *Fluviicola riflensis'*	C	CP022585	ASS49886	>512
* E. coli* pUA1108::*folA Alcanivorax pacificus*	C	NZ_CP004387	WP_008736147	32
* E. coli* pUA1108::*folA Alcanivorax borkumensis*	C	AM286690	CAL17791	16
* E. coli* pUA1108::*folA Bacillus mobilis*	C	NZ_CP031443	WP_000637217	>512
* E. coli* pUA1108::*folA Ralstonia solanacearum*	C	NC_003295	WP_011000898	0.5
* E. coli* pUA1108::*folA* blood disease bacterium A2-HR MARDI	M	CP019912	AQW32254	1
* E. coli* pUA1108::*folA E. coli* O104:H4	M	CP003298	AFS59762	2

Two remaining clades of novel mobile DHFR homologues from clinically relevant bacteria associated with chromosomal FolA proteins were shown to confer resistance to trimethoprim on *E. coli* (Table 1). To investigate whether the sequence determinants conferring resistance had originated in the associated chromosomal background, we cloned the most closely related chromosomal *folA* gene as well as a gene encoding an additional DHFR homologue from the same genus, and performed broth microdilution assays to determine the MIC of trimethoprim. We also performed ancestral state reconstruction of the molecule encoding the DHFR homologues (chromosomal/mobile trait), as determined during the data collection process (Tables S5 and S12).

The combined results of Table 1 and Fig. 1 reveal different patterns of trimethoprim-resistance acquisition. The protein KMV08986 is a DHFR homologue harboured by a conjugative plasmid from an *Acinetobacter baumannii* clinical isolate. Its most closely related chromosomally encoded DHFR homologue is the FolA protein of *Flavobacterium branchiophilum *, which confers resistance to trimethoprim (Table 1). To ascertain whether this chromosomally encoded DHFR homologue was encoded by a bona fide *folA* gene, instead of a mobile *dfrA* gene that integrated into the chromosome, we compared the genus-wide distribution of pairwise amino acid sequence alignment distances between FolA proteins to the pairwise distance of the identified homologue versus all other FolA proteins in the genus. The *Flavobacterium branchiophilum* FolA sequence is significantly different from other *Flavobacterium* FolA sequences (Mann–Whitney U *P*<0.05; Table S13), raising the possibility that this chromosomal gene could be in fact a recombined mobile *dfrA* gene. However, phylogenetic analysis with a broader representation of *Flavobacterium* sequences (Fig. S3) confirms the well-supported branching of *Flavobacterium branchiophilum* FolA with other *Flavobacterium* species FolA proteins, and comparative genomics analysis reveals that the genetic neighbourhood of the chromosomal *folA* gene is conserved in the genus *Flavobacterium* (Fig. S4). Furthermore, the FolA protein of a prototypical genus member, *Flavobacterium faecale *, also confers resistance to trimethoprim on *E. coli* (Table 1). These results indicate that the FolA protein was likely resistant to trimethoprim in the ancestor of extant *Flavobacterium* species, which diverged more than 50 million years ago [53]. The branching of the *Acinetobacter baumannii* protein KMV08986 in the reconstructed phylogeny and the associated ancestral state reconstruction indicates that this mobile DHFR homologue likely originated via mobilization of a chromosomal *folA* gene within the phylum *Bacteroidetes *. The encoded FolA protein was likely resistant to trimethoprim, but the exact donor species remains to be elucidated.

In contrast to *Flavobacterium* proteins, the *Acinetobacter schindleri* FolA protein does not confer resistance to trimethoprim, in agreement with previous reports of *Acinetobacter schindleri* susceptibility to trimethoprim [54], and with the well-established susceptibility of *Acinetobacter baumannii* FolA to trimethoprim [55, 56]. The *Acinetobacter schindleri* FolA protein is closely related to three mobile DHFR homologues conferring resistance to trimethoprim and harboured by *Acinetobacter baumannii* (protein ID: WP_031380727, WP_034702334) and *Acinetobacter defluvii* (protein ID: WP_004729503) clinical and environmental isolates. These mobile DHFR homologues branch within a well-supported clade of chromosomal *Acinetobacter* FolA proteins, as supported by ancestral state reconstruction (Fig. 1, Table S12). The trimethoprim susceptibility of *Acinetobacter* chromosomal *folA* genes and the phylogenetic placement of these DHFR homologues, hence, indicates that the observed resistance to trimethoprim was acquired immediately prior to or after mobilization from an *Acinetobacter* chromosomal background. This is supported by the observation that these mobile DHFR homologues confer different levels of resistance to trimethoprim (Table 1), and that the largest MIC correlates with the location of the DHFR homologue on a plasmid harbouring multiple antibiotic-resistance determinants (Fig. S5). The gene encoding this DHFR homologue is preceded by an insertion sequence transposase (Fig. S5), in an arrangement that has been reported to drive up expression of the DHFR homologue through promoter enhancement [57]. However, the MIC determined here corresponds to that of the isolated DHFR ORF, indicating that it confers heightened resistance irrespective of the promoter driving its expression. This suggests that these DHFR homologues have acquired mutations conferring heightened resistance to trimethoprim in parallel to their broader dissemination on multi-resistant mobile elements. Based on their validated trimethoprim-resistance phenotype and their level of amino acid sequence identity versus previously reported DfrA proteins (<95 %; Table S14) [13], we propose to designate these *Acinetobacter* DHFR homologues as DfrA38 (protein ID: KMV08986), DfrA39 (protein ID: WP_031380727), DfrA40 (protein ID: WP_034702334) and DfrA41 (protein ID: WP_004729503).

Here, we report the identification of trimethoprim-susceptible chromosomal *folA* genes that are closely related to mobile *dfrA* genes, as well as the discovery of chromosomally encoded *folA* genes conferring resistance to trimethoprim. This indicates that, in contrast to sulfonamides [25], trimethoprim-resistance mutations with small or negligible fitness cost must occur frequently enough in natural environments. These *folA* variants can then be selected for and mobilized upon exposure to trimethoprim. It is well-documented that resistance to trimethoprim, mediated by mutations in the chromosomal *folA* gene, develops very rapidly and in a fairly structured way [58–60], whereas resistance to sulfonamides takes much longer to evolve in a laboratory setting. Moreover, sulfonamide-resistant mutants typically show significantly reduced affinity to PABA. This results in a net fitness cost in the absence of sulfonamide that is only palliated by the emergence of subsequent compensatory mutations [61, 62]. Beyond structural constraints on the respective binding pockets, a crucial difference between both chemotherapeutic agents lies in their respective targets. While trimethoprim directly inhibits DHFR, sulfonamides compete with PABA for access to DHPS, yielding a non-productive sulfonamide-bound di-hydropterin. For sulfonamides, therefore, it is the PABA-to-sulfonamide ratio that limits the production of di-hydropteroate from a limited pool of pteridine di-phosphate, and this cannot be altered via overexpression of DHPS [63]. Conversely, DHFR overexpression can provide partial resistance to trimethoprim, and mutations enhancing DHFR expression have been reported to be the first to appear in directed evolution experiments [60]. The ability to obtain partial resistance through overexpression may provide a stepping stone for the gradual accumulation and refinement of mutations conferring substantial resistance with little fitness cost and, hence, facilitate the development of trimethoprim resistance [59, 60].

### Trimethoprim resistance in chromosomally encoded *folA* genes

Besides uncovering novel *dfrA* genes, the phylogenetic analysis in Fig. 1 also identifies several chromosomal *folA* genes associated with previously reported *dfrA* genes. Two of these chromosomal *folA* genes have already been reported in the literature as putative origins of *dfrA* genes, and their identification here provides some degree of validation for the phylogenetic approach implemented in this work. The putative chromosomal origin for *Staphylococcus aureus* Tn*4003* S1-DHFR has been identified as the chromosomally encoded *dfrC* gene (* Staphylococcus epidermidis *) and is reported to be susceptible to trimethoprim [64]. The *Enterococcus faecalis dfrE* gene, identical to the chromosomally encoded *folA* gene of *Enterococcus faecalis *, was reported to confer moderate resistance to trimethoprim in *E. coli *, but only when cloned in a multicopy plasmid, which could easily result in overexpression-mediated resistance [63, 65].

To ascertain whether the chromosomal *folA* genes found here to be associated with other known *dfrA* genes (*dfrA20*, *dfrA26* and the *dfrDGK* cluster) confer resistance to trimethoprim, we performed broth microdilution assays to determine the MIC of trimethoprim on these chromosomally encoded FolA proteins and on another FolA protein from the same genus. In all cases, both related FolA proteins confer resistance to trimethoprim (Table 1). The most closely associated chromosomal *folA* genes are not significantly different from other *folA* genes in their respective genera (Mann–Whitney U *P*>0.05; Table S13), as reflected also by substantial conservation of the *folA* genomic neighbourhood (Fig. S4). Together, these data indicate that resistance to trimethoprim was present in the ancestor of these genera. The *dfrA26* gene was identified in a *K. pneumoniae* clinical isolate and its most closely associated chromosomal *folA* gene is a member of the genus *Alcanivorax *. The branching pattern of *dfrA26* within this clade and ancestral state reconstruction results (Fig. 1, Table S12) suggest that it arose via mobilization of a chromosomal *folA* gene from the genus *Alcalinivorax*. The *dfrDGK* genes have been reported in *Enterococcus faecalis *, *Enterococcus faecium* and *Staphylococcus aureus *, and ancestral state reconstruction results indicate that these mobile *dfrA* genes originated through mobilization of a member of closely related genus *Bacillus *, members of which have been reported to be naturally resistant to trimethoprim [66]. In both cases, therefore, the phylogenetic evidence and the similarity in mol% G+C content among chromosomal and mobile genes (Fig. 1, Table S15) point towards a mobilization event that has to date remained circumscribed to related genera. Conversely, the *dfrA20* gene was identified in a *Pasteurella multocida* isolate, yet the chromosomal *folA* gene most closely associated to it is encoded by *Fluviicola taffensis *, a *Bacteroidetes *; hence, suggesting a much more distant mobilization event (Fig. 1, Table S15). In all three cases, however, we find evidence that pre-existing resistant *folA* genes can be readily mobilized from both close (e.g. *dfrDGK*) or distant (e.g. *dfrA20*) species.

**Fig. 3.**
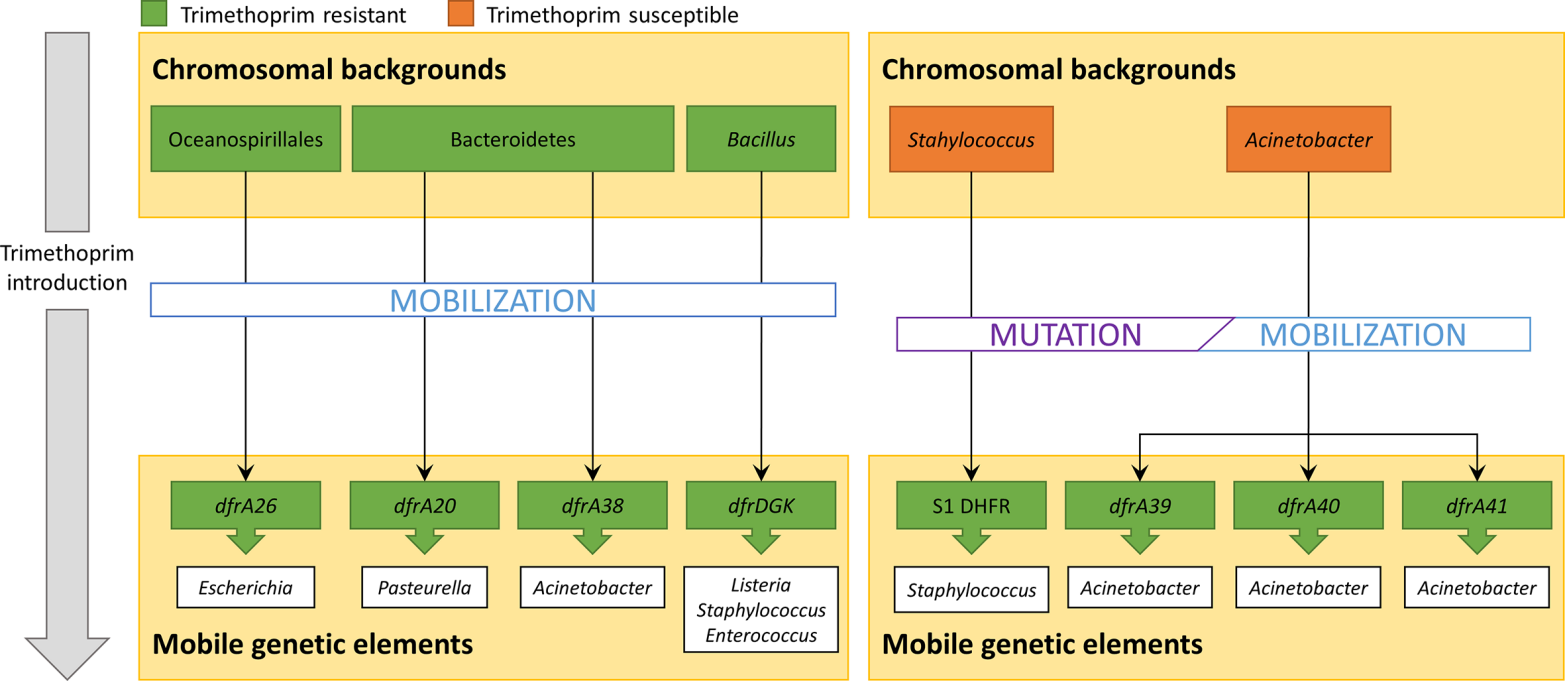
Schematic representation of the two proposed evolutionary processes (based on the results presented in Figs 1 and 2, and Table 1) leading to the dissemination of trimethoprim-resistance determinants. Left panel: upon the introduction of trimethoprim, mobilization events involving pre-existing resistant chromosomal *folA* genes can be favourably selected. Right panel: following the introduction of trimethoprim, mobilization events involving *folA* genes with novel mutations that confer resistance to this chemotherapeutic agent may be selected for and disseminated among closely related bacteria.

The resistance to trimethoprim reported here for the chromosomal *folA* genes of two different genera of *Bacteroidetes *, two distinct *Alcanivorax* species and a *Bacillus* strain underscores the deep ancestry of chromosomal mutations yielding resistance to trimethoprim. The *folA* genes of *Flavobacterium* and *Fluviicola* were shown here to confer resistance to trimethoprim. These two genera are thought to have diverged more than 500 million years ago and define major lineages within the *Flavobacteriales *, suggesting that resistance to trimethoprim emerged in an ancestor of this bacterial order. It is worth noting that several of the chromosomal *folA* genes shown here to be associated with mobile DHFR homologues (* Alcanivorax *, *Flavobacterium* and *Fluviicola *) appear to be resistant at the genus level and correspond to genera of aquatic bacteria. This parallels our recent identification of soil and subterranean water bacteria as the likely originators of clinical sulfonamide-resistance genes [25], and suggests that the intensive use of trimethoprim/sulfamethoxazole in agriculture, aquaculture and animal husbandry in the last 50 years may have acted as a trigger for the selection and mobilization of pre-existing *folA* and *folP* genes conferring resistance to trimethoprim and sulfonamides. Conversely, trimethoprim-susceptible chromosomal *folA* genes found here to be associated with *dfrA* genes belong to clinically relevant genera (* Staphylococcus* and *Acinetobacter *) that may have been under more direct trimethoprim pressure. This suggests that among relatively isolated bacterial populations, frequent exposure to high levels of trimethoprim may trigger the mobilization of spontaneous *folA* mutants, whereas longer term exposure to sub-lethal doses of trimethoprim in ecological rich habitats might instead rely predominantly on the mobilization of naturally resistant *folA* genes (Fig. 3).

### Phage-encoded *folA* genes do not confer resistance to trimethoprim

Our phylogenetic analysis also identifies a well-defined clade of *Enterobacteriaceae* cryptic plasmids derived from *Salmonella* phage SSU5 and encoding DHFR homologues [67–70]. Genes encoding DHFR homologues occur frequently in many bacteriophage families, often in tandem with thymidylate synthase genes [71], but their functional role has not been fully elucidated. We performed broth microdilution assays to determine the MIC of trimethoprim of *E. coli* O104:H4 DHFR (protein ID: AFS59762). This phage-encoded DHFR does not confer resistance to trimethoprim (Table 1). The high amino acid sequence identity and neighbourhood conservation among the DHFR enzymes encoded by these *Enterobacteriaceae* cryptic plasmids and phages (Table S16, Fig. S4) would presumably suggest that these DHFR enzymes are susceptible to trimethoprim.

Bacteriophages can transfer substantial amounts of genetic material via generalized transduction, and their potential as reservoirs of antibiotic-resistance determinants has gained increased attention with the advent of metagenomics [72, 73]. However, recent studies have shown that many potential resistant determinants encoded by phages do not confer resistance against their putative targets. Furthermore, only a small proportion of complete phage genomes contain putative antibiotic-resistance genes [74]. Enzymes participating in the folate biosynthesis pathway, however, are relatively frequent in phage genomes. These include homologues of the *folP* gene encoding DHPS, of the *thyX* gene encoding flavin-dependent thymidylate synthase [75–77] and, predominantly, homologues of the *folA* gene encoding DHFR, often found in tandem with the *thyA* gene encoding type 1 thymidylate synthase [71].

Early work on *Enterobacteria* phage T4 showed that the phage-encoded *thyA* and *folA* gene products are functional and also participate in the phage baseplate structure [78], and *thyX* has been shown to be functional in a number of phages [75–77]. It has been proposed that these genes help bacteriophages overcome shortages in the deoxynucleotide pool during replication, but their potential in conferring resistance to sulfonamides or trimethoprim remains largely unexplored. The detection here of DHFR homologues in *Enterobacteriaceae* cryptic plasmids and phages, and the subsequent assessment of their trimethoprim susceptibility, reinforces the notion that these genes have been functionally co-opted by phages principally for deoxynucleotide synthesis. Nonetheless, these genes may still confer partial trimethoprim resistance as a by-product of *folA* overexpression, as recently reported for *Stenotrophomonas maltophilia* phage DLP4 [79].

### Conclusions

Recent work has shown that resistance to sulfonamide, a synthetic chemotherapeutic agent, can be present in the bacterial pangenome well before the discovery of the agent. Here, we have used a combination of *in silico* and *in vitro* techniques to identify novel trimethoprim-resistance genes, and to identify chromosomal *folA* genes that are strongly associated with novel and previously reported *dfrA* genes. We find that most of the chromosomal *folA* genes associated with mobile *dfrA* genes confer resistance to trimethoprim, but we detect cases of novel mutations being rapidly mobilized. Hence, our work shows that the observations from sulfonamide resistance extend to trimethoprim, with generalized chromosomal resistance determinants predating the origin of several genera and several clusters of resistance genes disseminated broadly among clinical isolates. Moreover, this work also reveals that, unlike sulfonamides, resistance to trimethoprim is relatively easy to generate and frequently associated with species from the same clade it originated in. The identification of ancient resistance determinants for two synthetic chemotherapeutic agents strongly suggests that resistance to any novel drugs is likely to be already present in the bacterial pangenome. Systematic screening of existing natural variants could provide, therefore, the means to pre-emptively identify derivatives presenting widely distributed natural resistance determinants and, conversely, to engineer derivatives that circumvent most, if not all, natural resistant variants.

## Supplementary Data

Supplementary material 1

Supplementary material 2
