# Supplementary material for: Exploration into the origins and mobilization of di-hydrofolate reductase genes and the emergence of clinical resistance to trimethoprim
Source: Microb Genom. 2020 Sep 24;6(11):mgen000440. doi: 10.1099/mgen.0.000440 (PMC7725336; doi:10.1099/mgen.0.000440)
Supplement: Supplementary material 1 [file mgen-6-440-s001.pdf]

```
                  10        20        30        40        50        60        70
          ....|....|....|....|....|....|....|....|....|....|....|....|....|....|
dfrA34    -----------------MITACVA-IDSDGGFGAQGTLPWA-IPEEFAFYQEHVRG-GICIIGGRSFNDL
dfrA9     ---------------MASLNMIVA-VNKTGGIGFENQIPWH-EPEDLKHFKAVTMN-SVLIMGRKTFASL
dfrA8     ---------------MIELHAILA-ATANGCIGKDNALPWPPLKGDLARFKKLTMG-KVVIMGRKTYESL
dfrA24    ----MTYQLDVSKILSFDLEAIVA-ATENGGIGYKGDLPWR-LQGDLKRFREITQG-GIVIMGAGTYKSL
dfrA3b    ----------------MTKQAIFA-VAENLAFGLGGGLPWDTLKDDLQFFKRLTEG-TDLVMGASTYRTL
dfrA10    ----------------MNISLIFANELITRAFGNQGKLPWQFIKEDMQFFQKTTEN-SVVVMGLNTWRSL
dfrA17    ----------------MKISLISA-VSENGVIGSGPDIPWS-VKGEQLLFKALTYN-QWLLVGRKTFDSM
dfrA32    ----------------MKISLISA-VSENGVIGSGPDIPWS-AKGEQLIFKALTYN-QWLLVGRKTFDSM
dfrA7     ----------------MKISLISA-TSENGVIGNGPDIPWS-AKGEQLLFKALTYN-QWLLVGRKTFDSM
dfrA15    ----------------MKLSLMAA-ISKNGVIGNGPDIPWS-AKGEQLLFKAITYN-QWLLVGRKTFESM
dfrA15b   ----------------MKLSLMAA-ISKNGVIGNGPDIPWS-AKGEQLLFKAITYN-QWLLVGRKTFESM
dfrA1     ----------------MKLSLMVA-ISKNGVIGNGPDIPWS-AKGEQLLFKAITYN-QWLLVGRKTFESM
dfrA28    ---------------------MVA-ISKNGVIGNGPDIPWS-AKGEQLLFKAITYN-QWLLVGRKTFESM
dfrA16    ----------------MKLSLMAA-KSKNGIIGNGPDIPWS-AKGQQLLFRAIIYN-QWLLVGRKTFESM
dfrA16b   ----------------MKLSLMAA-KSKNGIIGNGPDIPWS-AKGEQLLFKAITYN-QWLLVGRKTFESM
dfrA5     ----------------MKVSLMAA-KAKNGVIGCGPHIPWS-AKGEQLLFKALTYN-QWLLVGRKTFESM
dfrA30    ----------------MKVSLMAA-RARNGVIGCGPHIPWS-AKGEQLLFKALTYN-QWLLVGRKTFESM
dfrA14    ----------------MKVSLIAA-KRKNGVIGCGPDIPWS-AKGEQLLFKALTYN-QCLLVGRKTFESM
dfrA25    ---------------------MAA-RAKNGVIGCGPDIPWS-AKGEQLLFKALTYN-QWLLVGRKTFESM
dfrA29    ---------------------MAA-RAKNGVIGCGPNIPWS-AKGEQLLFKALTYN-QWLLVGRKTFESM
dfrA27    ----------------MKISLMAA-KARNGVIGCGSDIPWN-AKGEQLLFKAITYN-QWLLVGRKTFEAM
dfrA6     ----------------MKISLMAA-VSENGVIGSGLDIPWH-VQGEQLLFKAMTYN-QWLLVGRKTFDSM
dfrA31    ----------------MKISIMAA-VSENGVIGSGLDIPWH-VQGEQLLFKAMTYN-HWLLVGRKTFDSM
dfrA13    -----------MNPESVRIYLVAA-MGANRVIGNGPDIPWK-IPGEQKIFRRLTES-KVVVMGRKTFESI
dfrA21    -----------MNPESVRIYLVAA-MGANRVIGNGPDIPWK-IPGEQKIFRRLTES-KVVVMGRKTFESI
dfrA22    -----------MNPELVRIYLVAA-MGANRVIGNGPDIPWK-IPGEQKIFRRLTEG-KVVVMGRKTFESI
dfrA33    -----------MNPESVRIYLVAA-MGANRVIGNGPDIPWN-IPGEQKIFRRLTEG-KVVVMGRKTFESI
dfrA12    -----------MNSESVRIYLVAA-MGANRVIGNGPNIPWK-IPGEQKIFRRLTEG-KVVVMGRKTFESI
dfrF      -----------------MIGLIVA-RSKNNVIGKNGNIPWK-IKGEQKQFRELTTG-NVVIMGRKSYEEI
dfrA3     ----------------MLISLIAA-LAHNNLIGKDNLIPWH-LPADLRHFKAVTLG-KPVVMGRRTFESI
dfrA26    MADEEYDPLLDDDMEDAKVAVIAA-RAQNGCIGRHGKLPWK-LPGDLKYFRERTWG-KPIIMGRKTWESL
dfrG      ----------------MKVSLIAA-MDKNRVIGKENDIPWR-IPKDWEYVKNTTKG-HPIILGRKNLESI
dfrK      ----------------MKVSLIAA-MDKNRVIGKENDIPWR-IPEDWEYVKNTTKG-YPIILGRKNLESI
dfrD      ----------------MKISLIVA-MDKKRVIGKDNDIPWR-ISSDWEYVKNTTKG-HAIILGRKNLQSI
dfrA20    --------------MGIKYSLIVA-IGKHREMGADNDLLWH-LPRDMQFFKETTTG-HAVVMGRKSWESI
S1_dhfr   ----------------MTLSIIVA-HDKQRVIGYQNQLPWH-LPNDLKHIKQLTTG-NTLVMARKTFNSI
dfrE      -----------------MLAAIWA-QDEQGVIGKEGKLPWH-LPNDLKFFKEKTIH-NTLVLGRATFEGM
dfrA18    -------------MSHPQLELIVA-VDSKLGFGKGGKIPWK-CKEDMARFTRISKEIRVCVIGKHTYTDM
dfrA19    -------------MSHPQLELIVA-VDSKLGFGKGGKIPWK-CKEDMARFTRISKEIRVCVIGKHTYTDM
dfrA23    ---------------MPTVEIIVA-VDPVGGFGRNGQIPWT-CKEDMKRFTTISKEIRVCVMGKNTYKDM
dfrB5     ------------------------------MDQGRSEVSNP-VAGQFAFPSNAAFG-----MGDRVRKKS
dfrB7     ------------------------------MDQGSNEVGNP-VAGQFSFPSNAAFS-----MGDRVRKKS
dfrB6     ------------------------------MDQGSNEVINP-VAGQFASPSNATFG-----MGDRVRKKS
dfrB1     ----------MQRVVGPHRTPRSS-QERSEMERSSNEVSNP-VAGNFVFPSNATFG-----MGDRVRKKS
dfrB2     ------------------------------MGQSSDEANAP-VAGQFALPLSATFG-----LGDRVRKKS
dfrB3     ------------------------------MDQHNNGVSTL-VAGQFALPSHATFG-----LGDRVRKKS
dfrB4     ------------------------------MNEGKNEVSTS-AAGRFAFPSNATFA-----LGDRVRKKS
```

Figure S1 – Multiple sequence alignment including all reported mobile DHFR proteins. DfrB protein sequences are highlighted in yellow.

Figure S1 – (continued).

```
                         80        90       100       110       120       130       140
              ....|....|....|....|....|....|....|....|....|....|....|....|....|....|
dfrA34        VHLSLSPKGGLYKKCLLRTTPHIVVSSSHELVYDPSIMALIEADRRHLDLYFVNTVDAAVKLAKGLGG--
dfrA9         P---------------------KVLPGRLHVVVSKTVPPTQNTDQVVYVSTYQIAVRTASLLVDKP----
dfrA8         P---------------------VKLEGRTCIVMTRQALELPGVRDAN-GAIFVNNVSDAMRFAQEES---
dfrA24        P---------------------SPLKDRINIVITKKSEISWTACY---DVRVVNSPEDALRMVGRIIDEK
dfrA3b        P--------------------LLPTNNRQFIVVSNTEEPSLNVH-----VVSPEHFKAFLSKTSR-----
dfrA10        P--------------------KMKKLGRDFIVISSTITEHEVLNN---NIQIFKSFESFLEAFRD-----
dfrA17        G----------------------VLPNRKYAVVSKNGISSSNE-----NVLVFPSIENALKELSK-----
dfrA32        G----------------------VLPNRKYAVVSKNGISGSNE-----NVLVFPSIENALQELSK-----
dfrA7         G----------------------VLPNRKYAVVSRKGISSSNE-----NVLVFPSIEIALQELSK-----
dfrA15        G----------------------ALPNRKYAVVTRSSFTSSDE-----NVLVFPSIDEALNHLKT-----
dfrA15b       G----------------------ALPNRKYAVVTRSSFTSSDE-----NVLVFPSIDEALNHLKT-----
dfrA1         G----------------------ALPNRKYAVVTRSSFTSDNE-----NVLIFPSIKDALTNLKK-----
dfrA28        G----------------------ALPNRKYAVVTRSSLTSDNE-----NVVIFPSIKDALTNLKK-----
dfrA16        G----------------------ALPNRKYAVVTRSNFSTNDE-----GVMVFSSIQDALINLEE-----
dfrA16b       G----------------------ALPNRKYAVVTRSNFSTNDE-----GVMVFSSIQDALINLQE-----
dfrA5         G----------------------ALPNRKYAVVTRSAWTADND-----NVIVFPSIEEAMYGLAE-----
dfrA30        G----------------------ALPNRKYAVVTRSAWTANND-----NVVVFPSIEEAMGGLAK-----
dfrA14        G----------------------ALPNRKYAVVTRSGWTSNDD-----NVVVFQSIEEAMDRLAE-----
dfrA25        G----------------------PLPNRKYAVVTRSNWTAANE-----NVVVFPSIDEAMGRLGE-----
dfrA29        G----------------------PLPNRKYAVVTRSNWTAANE-----NVVVFPSIDEAMGRLGE-----
dfrA27        G----------------------ALPNRKYAVVSRSGSVATND-----DVVVFPSIEAAMRELKT-----
dfrA6         G----------------------KLPNRKYAVVTRSKIISNDP-----DVVYFASVESALAYLNN-----
dfrA31        G----------------------KLPNRKYAVVTRSEMVSNDP-----DVIYFTSIESALSYLDN-----
dfrA13        G---------------------KPLPNRHTVVLSRQAGYSAPG------CAVVSTLSHVSPSTAE-----
dfrA21        G---------------------KPLPNRHTVVLSRQARYSAPG------CAVVSTLSQAIAIAAE-----
dfrA22        G---------------------KPLPNRRTVVLSRQASYSAAG------CAVVSTLSQAIAIAAE-----
dfrA33        G---------------------KPLPNRRTVVLSRQASYSAAG------CAVVSTLSQAIAIAAE-----
dfrA12        G---------------------KPLPNRHTLVISRQANYRATG------CVVVSTLSHAIALASE-----
dfrF          G---------------------HPLPNRMNIVVSTTTEYQGDN------LVSVKSLEDALLLAK------
dfrA3         G---------------------RPLPGRRNVVVSRNPQWQAEG------VEVAPSLDAALALLTD-----
dfrA26        N---------------------GALPGRTNIVVTRQQGYEAEGARVVDSIEEAISLAQSIALIEA-----
dfrG          G---------------------RALPDRRNIILTRDKGFTFNG------CEIVHSIEDVFELCKN-----
dfrK          G---------------------RALPGRRNIILTRDKGFSFNG------CEIVHSIEDVFELCNS-----
dfrD          G---------------------RALPDRRNIILTRDKNFNFKD------CEIAHSIEAAFKLCEN-----
dfrA20        PQK------------------YRPLPNRLNFVLTRDKNYSAEG------ATVIYDLKEVAQHLEG-----
S1_dhfr       G---------------------KPLPNRRNVVLTNQASFHHEG------VDVINSLDEIKELS-------
dfrE          GC--------------------RPLPNRTTIVLTSNPDYQAEG------VLVMHSVEEILAYADK-----
dfrA18        RDMQLEKDG-----AEERIKEKGILPERESFVISSTLKQEDVIG-----ATVVPDLRAVINLYEN-----
dfrA19        RDMQLEKDG-----AEERIKEKGILPERESFVISSTLKQEDVIG-----ATVVPDLRAVINLYEN-----
dfrA23        LDMQMKKEG-----AEERIKEKGILPERESYVVSSTLKPEDVIG-----ATVVPDLRAVLNQYHD-----
dfrB5         G-----------------------------------AAWQG-----------------------------
dfrB7         G-----------------------------------AAWQG-----------------------------
dfrB6         G-----------------------------------AAWQG-----------------------------
dfrB1         G-----------------------------------AAWQG-----------------------------
dfrB2         G-----------------------------------AAWQG-----------------------------
dfrB3         G-----------------------------------AAWQG-----------------------------
dfrB4         G-----------------------------------AAWQG-----------------------------
```

Figure S1 – (continued).

```
                      150       160       170       180       190       200       210
                ....|....|....|....|....|....|....|....|....|....|....|....|....|....|
dfrA34          ---MHANKDIHFIGGKRIYDAGLD--YCDEVYTSILPAVYLN-CDTFFPVEKLSRMFTPELYKTIPNQ--
dfrA9           -----EYSQIFVIGGKSAYENLAA--YVDKLYLTRVQLNTQQ--DTELDLSLFKSWKLVSEVPTITEN--
dfrA8           -----VGDVAYVIGGAEIFKRLAL--MITQIELTFVKRLYEGD-TYVDLAEMVKDYEQNGMEEHDLHT--
dfrA24          EEQGRDRPRVFVIGGASIYQALMP--FVSTLHWTEVHVEQLPE-EIGLDTYIEDFLSLRGTSTPKRKS--
dfrA3b          --------NLTIIGGSSLLTVDILS-KMDKIIMTTVYGSFDA--DVYLPTEVVSYVTGKASNATLFNN--
dfrA10          -----TTKPINVIGGVGLLSEAIE--HASTVYMSSIHMVKPVHADVYVPVELMNKLYSDFKYPENILWVG
dfrA17          -----VTDHVYVSGGGQIYNSLIE--KADIIHLSTVHVEVEG--DIKFPIM-PENFNLVFEQFFMSNI--
dfrA32          -----ITDHVYISGGGQIYESLIE--KADIIHLSTIHVEVEG--DIKFPIL-PEGFNLVFEQFFVSNI--
dfrA7           -----ITDHLYVSGGGQIYNSLIE--KADIIHLSTVHVEVEG--DINFPKI-PENFNLVFEQFFLSNI--
dfrA15          -----ITDHVIVSGGGEIYKSLID--KVDTLHISTIDIEPEG--DVYFPEI-PSSFRPVFSQDFVSNI--
dfrA15b         -----ITDHVIVSGGGEIYKSLID--KADTLHISTIDIEPEG--DVYFPEI-PGSFRPVFSQDFVSNI--
dfrA1           -----ITDHVIVSGGGEIYKSLID--QVDTLHISTIDIEPEG--DVYFPEI-PSNFRPVFTQDFASNI--
dfrA28          -----ITDHVIVSGGGEIYKSPID--QVDTLHISTIDIEPEG--DVYFPES-PAILG-QFYPRLRSNI--
dfrA16          -----ITDHVIVSGGGEIYKSLIS--KVDTLHISTVDIERDG--DIVFPEI-PDTFKLVFEQDFESNI--
dfrA16b         -----ITDHVIVSGGGEIYKSLIS--KVDTLHISTVDIERDG--DIVFPEI-PDTFKLVFEQDFESNI--
dfrA5           -----LTDHVIVSGGGEIYRETLP--MASTLHISTIDIEPEG--DVFFPNI-PNTFEVVFEQHFSSNI--
dfrA30          -----LNGHVIVSGGGEIYRETLP--MASTLHVSTIDIEPEG--DVFFPNI-PNFFEVVFEQHFSSNI--
dfrA14          -----FTGHVIVSGGGEIYRETLP--MASTLHLSTIDIEPEG--DVFFPSI-PNTFEVVFEQHFTSNI--
dfrA25          -----ITDHVIVAGGGEIYHETIP--MASTLHVSTIDVEPEG--DVFFPNI-PGKFDVVFEQQFTSNI--
dfrA29          -----ITDHVIVAGGGEIYHETIP--MASTLHVSTIDVEPEG--DVFFPNI-PGKFDVVFEQQFTSNI--
dfrA27          -----LTNHVVVSGGGEIYKSLIA--HADTLHISTIDSEPEG--NVFFPEI-PKEFNVVFEQELHSNI--
dfrA6           -----ATAHIFVSGGGEIYKALID--QADVIHLSVIHKHISG--DVFFPPV-PQGFKQTFEQSFSSNI--
dfrA31          -----TTTHVFVSGGGEIYKALIE--QADVIHLSVIHKHISG--DVFFPSV-PQSFKQTFEQSFSSNI--
dfrA13          -----HGKELYVARGAEVYALALP--HANGVFLSEVHQTFE-G-DAFFPVLNAAEFEVVSSETIQGTI--
dfrA21          -----HGKELYVAGGAEVYALALP--HANGVFLSEVHQTFE-G-DAFFPVLNAAEFEVVSSETIQGTI--
dfrA22          -----HGKELYVAGGAEVYALALP--RADGVFLSEVHQTFE-G-DAFFPVLDEAEFEVVSAETVQATI--
dfrA33          -----HGKELYVAGGAEVYALALP--RADGVFLSEVHQTFE-G-DAFFPALDAAEFDVVSAETVQATI--
dfrA12          -----LGNELYVAGGAEIYTLALP--HAHGVFLSEVHQTFE-G-DAFFPMLNETEFELVSTETIQAVI--
dfrF            ------GRDVYISGGYGLFKEALQ--IVDKMYITEVDLNIEDG-DTFFPEFDINDFEVLIGETLGEEV--
dfrA3           -----C-EEAMIIGGGQLYAEALP--RADRLYLTYIDAQLNG--DTHFPDYLSLGWQELERSTHPADD--
dfrA26          -----V-DEIMVLGGGEIYTQALP--QADILYLTEVHASVDG--DAFFPDVDLSQYQETQRQDFEPSG--
dfrG            -----E-EEIFIFGGEQIYNLFFP--YVEKMYITKIHHEFEG--DTFFPEVNYEEWNEVFAQKGIKND--
dfrK            -----E-EEIFIFGGEQIYNLFLP--YVEKMYITKIHYEFEG--DTFFPEVNYEEWNEVSVTQGITNE--
dfrD            -----E-EEVFIFGGEQIYVMFLP--YVEKMYVTKIHHEFEG--DTFFPVVNFDDWKEVSVEKGIKDE--
dfrA20          -----KNLTCFIIGGAQIYQLALETGLLNEMYVTQVHNTFEEA-DTFFPFVNWGEWEEEDILEQDKDE--
S1_dhfr         -------GHVFIFGGQTLYEAMID--QVDDMYITVIDGKFQG--DTFFPPYTFENWEVESSVEGQLDE--
dfrE            -----YEGVTVIGGGSVVFKELIP--ACDVLYRTMIHETFEG--DTFFPEIDWSVWEKVATVPGVVDE--
dfrA18          -----TDQRIAVIGGEKLYIQALS--SATKLHMTIIPREFDCD-RFIPVDPIQNNFHIDSSASETVEAT-
dfrA19          -----TDQRIAVIGGEKLYIQALS--SATKLHMTIIPREFDCD-RFIPVDPIQNNFHIDSSASETVEAT-
dfrA23          -----SDQRIAVIGGEKLYVQALA--SATKVHMTVMHKPYNCD-RTLPMSYIDKKF-VAGQGSITIQTA-
dfrB5           ----------QIVG---WYCTKLT----PEGYAVESEAHPGS--VQIYPVAALERIN-------------
dfrB7           ----------QIVG---WYCTKLT----PEGYAVESEAHPGS--VQIYPVAALERINGVQG---------
dfrB6           ----------QIVG---WYSTKLT----PEGYAVESEAHPGS--VQIYPVAALERVN-------------
dfrB1           ----------QIVG---WYCTNLT----PEGYAVESEAHPGS--VQIYPVAALERIN-------------
dfrB2           ----------QVVG---WYCTKLT----PEGYAVESESHPGS--VQIYPVAALERVA-------------
dfrB3           ----------QVVG---WYCTKLT----PEGYAVESESHPGS--VQIYPVAALERVA-------------
dfrB4           ----------RIVG---WYCTTLT----PEGYAVESESHPGS--VQIYPMTALERVA-------------
```

Figure S1 – (continued).

```
                   220       230       240
           ....|....|....|....|....|....|....
dfrA34     ----VHADIPVIKWTRKRA---------------
dfrA9      -KTKLIFQIWINPNPISEEPTC------------
dfrA8      ---YFTYRKKELTE--------------------
dfrA24     ---NLVLPPTPTTP--------------------
dfrA3b     --SDAKMAVYYG----------------------
dfrA10     DPIDSVYSLSIDKFVRPASLVGVPNDINT-----
dfrA17     ---NYTYQIWKKG---------------------
dfrA32     ---NYTYQIWKKG---------------------
dfrA7      ---NYTYQIWKKG---------------------
dfrA15     ---NYSYQIWQKG---------------------
dfrA15b    ---NYSYQIWQKG---------------------
dfrA1      ---NYSYQIWQKG---------------------
dfrA28     ---NYSYQIWQRVQCSTIRNCPLCTKRQVASLAG
dfrA16     ---NYCYQIWQKS---------------------
dfrA16b    ---NYCYQIWQKS---------------------
dfrA5      ---NYCYQIWQKG---------------------
dfrA30     ---NYCYQIWKKG---------------------
dfrA14     ---NYCYQIWKKG---------------------
dfrA25     ---NYCYQIWQKG---------------------
dfrA29     ---NYCYQIWQKG---------------------
dfrA27     ---NYRYQIWQRG---------------------
dfrA6      ---DYTYQIWAKG---------------------
dfrA31     ---DYTYQIWAKG---------------------
dfrA13     ---TYTHSVYARRNG-------------------
dfrA21     ---TYTHSVYARRNG-------------------
dfrA22     ---TYTHSVYARRNG-------------------
dfrA33     ---TYTHSVYARRNG-------------------
dfrA12     ---PYTHSVYARRNG-------------------
dfrF       ---KYTRTFYVRKNELSRFWI-------------
dfrA3      ---KNSYACEFVTLSRQR----------------
dfrA26     ---GNPYPFSFVVYQRT-----------------
dfrG       ---KNPYNYYFHVYERKNLLS-------------
dfrK       ---KNPYTYYFHIYERKAS---------------
dfrD       ---KNPYDYYFHIYERIR----------------
dfrA20     ---KHLYSFNIKKFTR------------------
S1_dhfr    ---KNTIPHTFLHLVRRKGK--------------
dfrE       ---KNLYAHDYETYHR-NDK--------------
dfrA18     -VDETQERIHFATYVRNNQ---------------
dfrA19     -VDETQERIHFATYVRNNQ---------------
dfrA23     -VDGETHPVKFITYERARP---------------
dfrB5      ----------------------------------
dfrB7      ----------------------------------
dfrB6      ----------------------------------
dfrB1      ----------------------------------
dfrB2      ----------------------------------
dfrB3      ----------------------------------
dfrB4      ----------------------------------
```

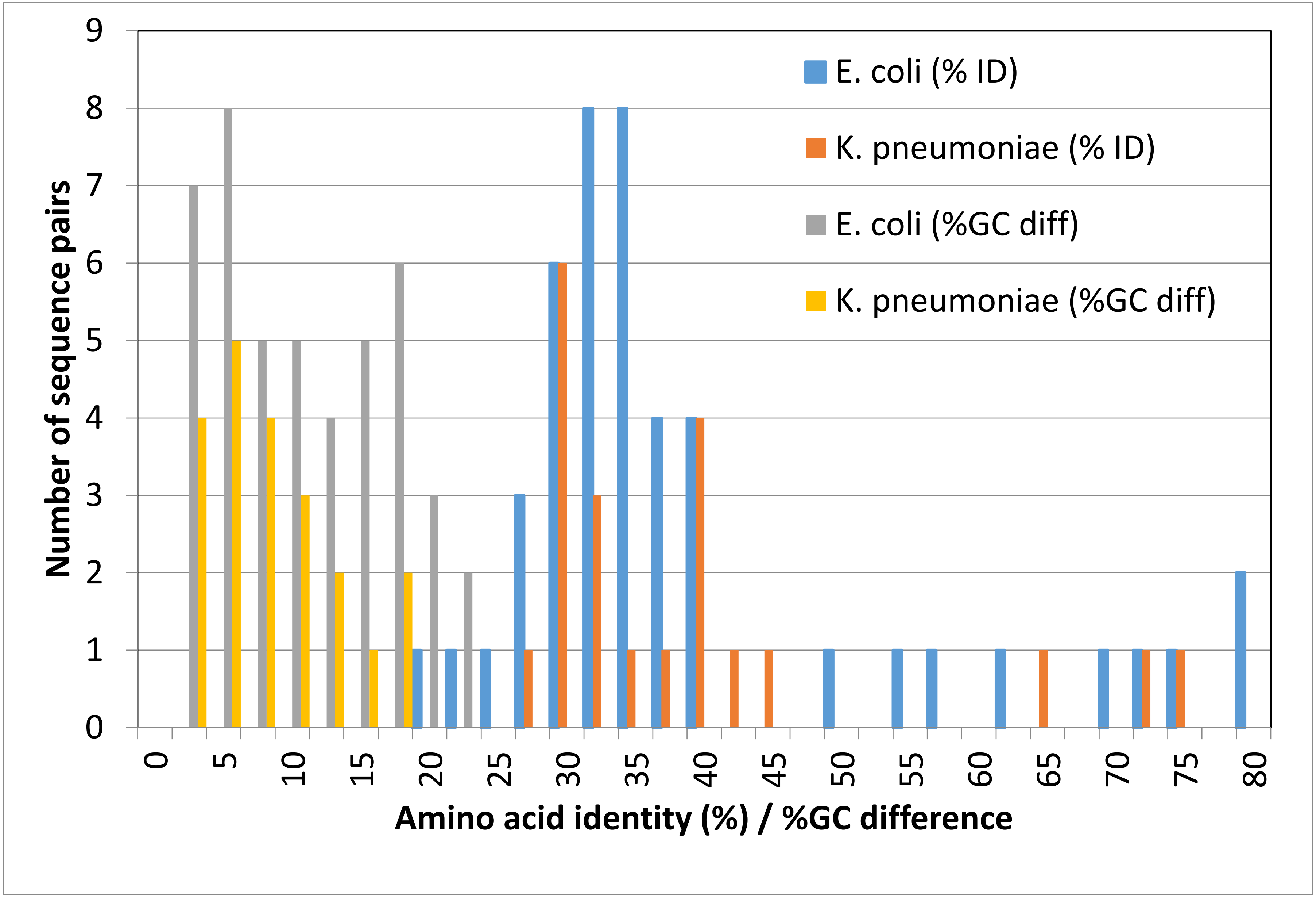

Figure S2 – Pairwise percent amino acid identity and %GC difference between aligned representative DfrA protein sequences harbored by mobile genetic elements of *E. coli* and *K. pneumoniae*.

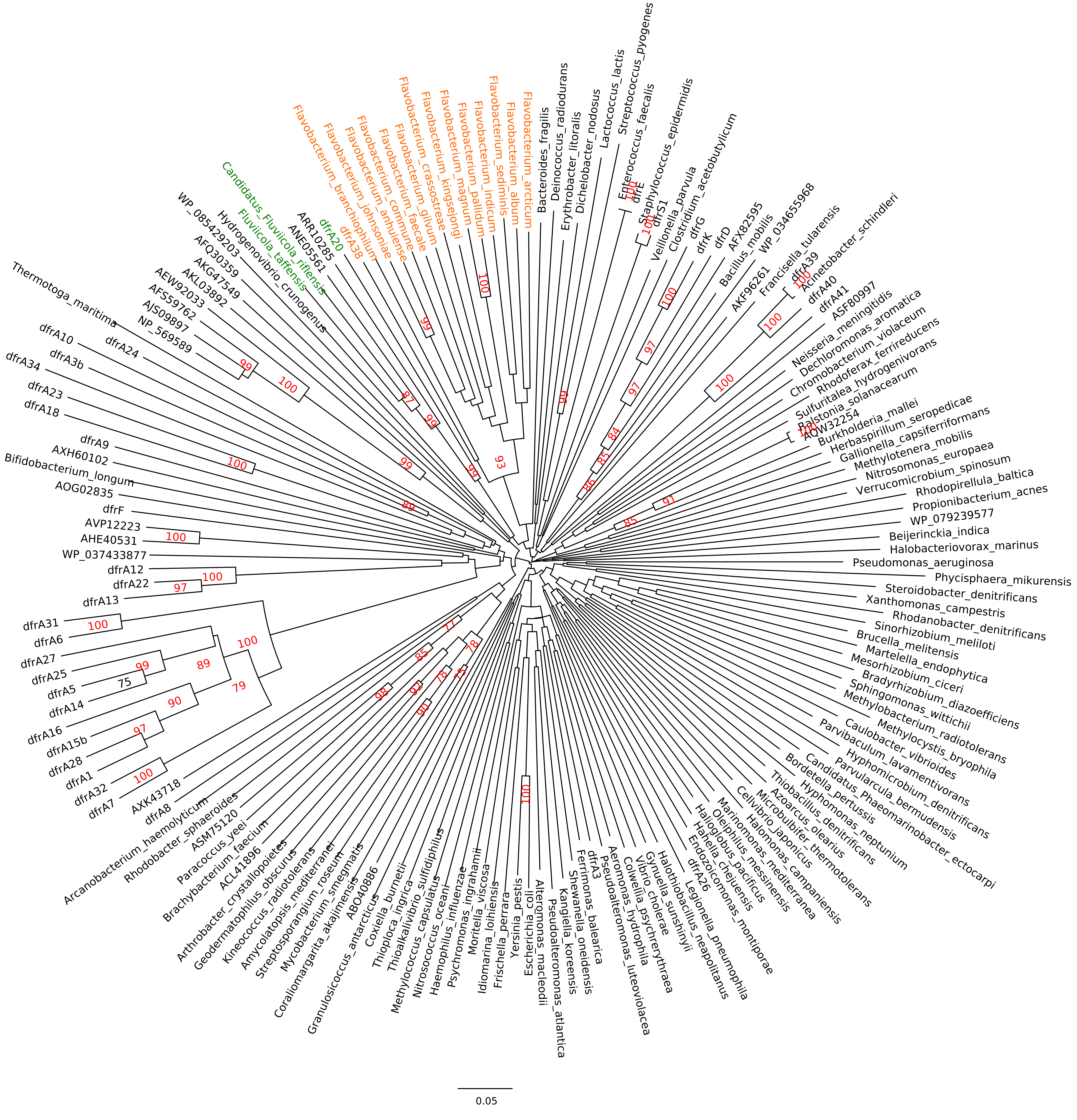

Figure S3 – Unrooted Neighbor-Joining tree of DHFR protein sequences. Branch support values are provided as the total number of 1000 bootstrap pseudo-replicates in which the branching was observed. Support values are only shown for branches with at least 75% bootstrap support. The DfrA20, DfrA38, *Fluviicola* and *Flavobacterium* DHFR protein sequences are highlighted.

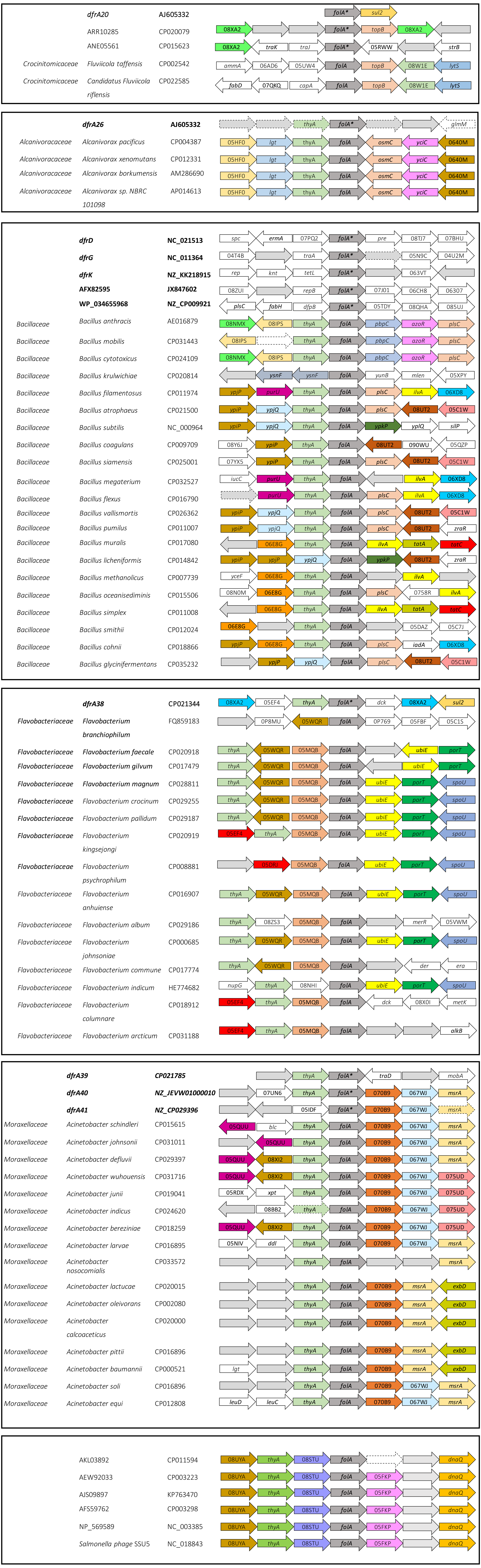

Figure S4 – Schematic representation of the genetic environment of mobile DHFR genes, their putative chromosomal origin and one representative complete genome assembly for each species within the corresponding genus. Arrow boxes indicate coding regions (discontinued arrows pinpoint pseudogenes). When available, gene names or NOG identifiers are provided. Color codes identify hits to the same NOG models.

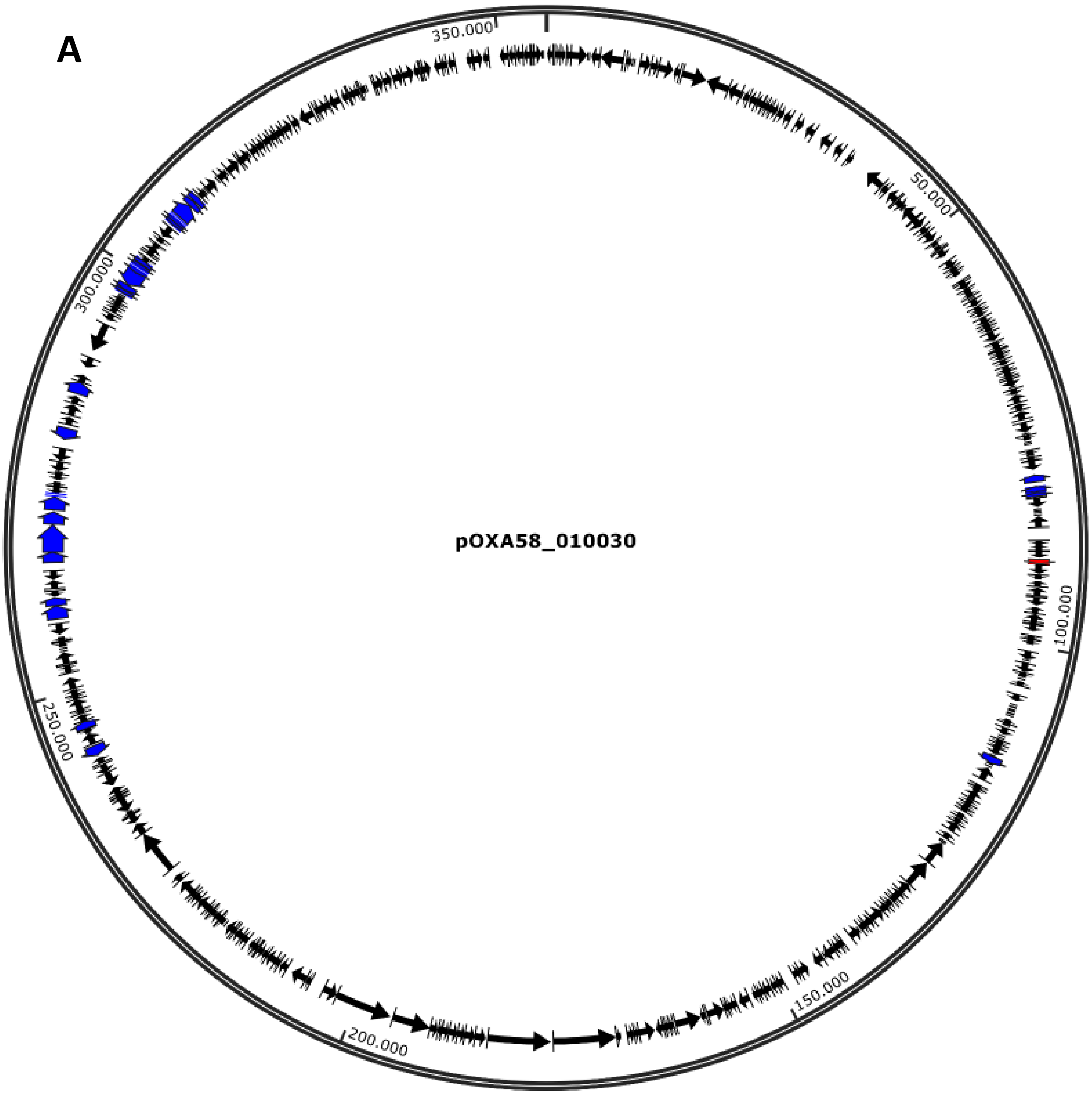

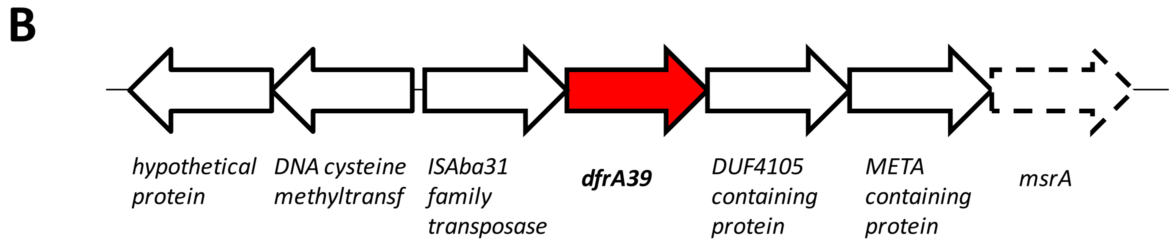

Figure S5 - (A) Graphical overview of *Acinetobacter defluvii* plasmid pOXA58_010030 with *dfrA41* (red arrow box) and other resistance determinants represented as blue arrow boxes.(B) Graphical overview of the genomic surroundings of the *dfrA41* gene in this plasmid. This figure was constructed using SnapGene Viewer.
